# Comparison of Environmental Microbiomes, Resistomes and Plasmidomes from a Human Tertiary Hospital and Companion Animal Veterinary Hospital in London, UK

**DOI:** 10.3390/antibiotics15060568

**Published:** 2026-06-02

**Authors:** Linzy Elton, Stuart Lutimba, Alonso Dupuy Mateos, Siân Marie Frosini, Rosanne Jepson, Alan Williams, Shanom Ali, Jelena Heaphy, Vicky Pang, Liam Commins, Conor O’Brien, Özge Yetiş, Estelle Caine, Imogen Ward, Monika Muzslay, Samuel Yui, Kush Karia, Ellinor Shore, Sylvia Rofael, Damien Mack, Claire Atkinson, Timothy D. McHugh, Emmanuel Q. Wey

**Affiliations:** 1 Centre for Innovation in Genomics and Microbiome Sciences, University of West London, London W5 5RF, UK; 2 UCL Centre for Clinical Microbiology, University College London, London NW3 2QG, UK; shanom.ali@nhs.net (S.A.); ozge.yetis@nhs.net (Ö.Y.);; 3 School of Applied and Health Science, London South Bank University, London SE1 0AA, UK; 4 Independent Veterinary Surgeon, London, UK; 5 Pathobiology and Population Sciences, Royal Veterinary College, Hawkshead AL9 7TA, UK; 6 Clinical Science and Services, Royal Veterinary College, Hawkshead AL9 7TA, UK; 7 Department of Infection Sciences, Health Services Laboratories, London WC1H 9AX, UK; 8 Environmental Research Laboratory, University College London Hospitals NHS Foundation Trust, London NW1 2PG, UK; estelle.caine@nhs.net (E.C.); imogen.ward3@nhs.net (I.W.); monika.muzslay@nhs.net (M.M.); samuel.yui@nhs.net (S.Y.); kush.karia@nhs.net (K.K.);; 9 Royal Free London Hospitals NHS Foundation Trust, London NW3 2QG, UK; 10 Faculty of Pharmacy, Alexandria University, Alexandria 21521, Egypt; 11 Department of Infection, Royal Free London NHS Foundation Trust, London NW3 2QG, UK

**Keywords:** metagenomics, One Health, Oxford Nanopore Technologies, veterinary, clinical, antimicrobial resistance, surveillance

## Abstract

**Background:** Human hospitals and veterinary centres are hotspots for resistant microbes and plasmids, and metagenomic sequencing offers an agnostic insight into microbiomes, resistomes, and mobilomes, informing strategies for reducing AMR spread. **Methods:** Environmental samples, including wastewater and surface swabs, were taken from a tertiary human hospital ward (36 samples) and a companion animal veterinary hospital (48 samples) in London. Whole DNA was extracted and metagenomic sequencing undertaken using Oxford Nanopore Technologies’ MinION. Data were analyzed for microbiomes, resistomes and mobilomes and compared. **Results:** Microbial diversity analyses highlight higher richness across human hospital (HH) environmental samples, but more evenness in veterinary hospital (VH) environmental samples. Diversity showed distinct microbial communities in the HH and VH samples. There were significantly more total antimicrobial resistance gene (ARG) types (*p* < 0.0001) in the environmental HH samples compared with the environmental VH samples. There was a significantly higher mean number of *Enterobacteriales* plasmid types (*p* ≤ 0.0001) in the HH samples. There were significantly more total Gram-Positive plasmid types (*p* ≤ 0.0001) in the VH samples. **Discussion:** This research highlights the presence of human and animal pathogens, ARGs and mobile genetic elements in clinical environments, underscoring the importance of multisectoral surveillance. Integrating taxonomic, resistome, and mobilome analyses provides a better understanding of the potential for AMR dissemination at the human–animal–environment interface. This provides insights relevant for the development of targeted surveillance and mitigation strategies within a OH framework.

## 1. Introduction

Antimicrobial resistance (AMR) is a critical global health challenge, threatening the effective treatment of infectious diseases across all One Health sectors [1,2]. The spread of AMR is driven by a complex interplay of horizontal gene transfer among microbial communities, the movement of resistant organisms across ecological boundaries, and the selective pressures from antimicrobial use in clinical, veterinary and agricultural settings [3,4,5,6,7].

The transmission of AMR is not limited to direct contact between humans and animals, but involves environmental compartments such as wastewater, soils, and surfaces across urban, agricultural and natural systems [8,9,10,11,12]. These environments may serve as both sinks and sources of AMR organisms and mobile genetic elements [2]. Understanding how plasmids, antimicrobial resistance genes (ARGs), and organisms differ between these different environments, and how they interact in shared environments, is critical for providing evidence for infection prevention and control (IPC) interventions to limit the spread of AMR [13].

Human hospitals (HHs) and veterinary hospitals (VHs) are particularly important places for selection pressure, which drives development of AMR, as they are both centres in which antimicrobial agents are routinely used. They are also both important junctions for potential AMR and multi-drug-resistant organism (MDRO) transmission, and we are becoming increasingly aware of the multi-directional risk factors associated with hospital inpatient stays and companion animal ownership [7,14,15,16,17,18,19]. Wastewater and environmental contamination from these settings may spread pathogens and ARGs within these settings and beyond into wider ecosystems, highlighting the need for integrated surveillance approaches [20,21].

Genomic technologies can provide enhanced information for the surveillance of AMR and pathogens. Traditional culture-based approaches can be less sensitive and may fail to capture the full diversity of microbial communities and ARGs [22,23,24]. Other molecular techniques, such as polymerase chain reaction (PCR), are more sensitive than phenotypic methods, but are limited in the specificity of the primers, probes and sequences targeted and may not distinguish between live and dead cells, thus preventing assignment of viable transmission/acquisition risk in clinical settings [25]. Genomic sequencing enables high-resolution characterization of pathogens, resistomes and plasmidomes. Furthermore, metagenomic analysis provides a culture-independent method to profiling microbial communities by sequencing total extracted DNA. This approach is valuable in One Health AMR surveillance, as the focus lies not only on pathogens of direct clinical concern but also on the wider resistome and mobilome contributing to the persistence and transmission of AMR [26,27]. The use of long read platforms such as Oxford Nanopore Technologies (ONT) are especially advantageous for metagenomics, as it allows higher-resolution differentiation between genetically similar species by spanning longer sections of genome [28].

Metagenomic sequencing has been increasingly employed to monitor AMR in wastewater, agriculture, and natural ecosystems, providing insights into both local and global dynamics [26,27,29,30,31,32]. However, multisectoral, comparative analyses of environmental resistomes remain limited in number. HHs present hubs for drug-resistant pathogens associated with human infections, while companion animal VHs are central to domestic animal health and may be a major site of antimicrobial consumption [33]. By comparing environmental metagenomes, resistomes and mobilomes from these two contexts we seek to detect similarities and differences in microbial community compositions, ARG prevalence, and plasmid diversity, which may reveal potential routes of cross-sectoral transmission [13].

This study aimed to use metagenomic sequencing to compare and contrast the species, ARGs and plasmids present in environmental samples collected from a companion animal VH and a tertiary HH in the same geographical area of London.

## 2. Results

### 2.1. Sample Recovery

**Figure 1:**
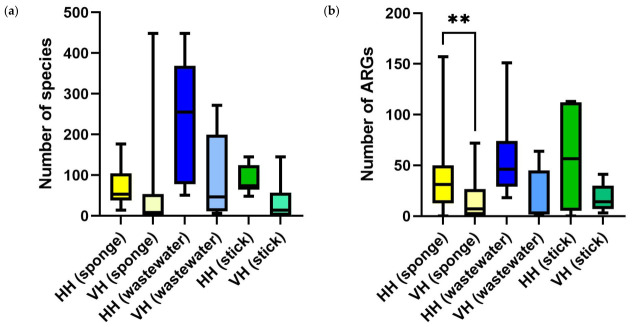
(**a**) The number of species and (**b**) number of ARGs were compared between the HH and VH samples and statistically analyzed using a *t*-test, ** = *p* = 0.0080. There was no significant difference between the HH and VH samples except for sponge swabs when identifying ARGs.

A total of 36 clinical and 48 metagenomic VH environmental samples were sequenced. For the HH environmental samples, 17/36 (47.2%) were sponge swabs, 6/36 (16.6%) were stick swabs and 13/36 (36.1%) were wastewater samples. For the VH samples 33/48 (68.7%) were sponge swabs, 7/48 (14.5%) were stick swabs, 5/48 (10.4%) were wastewater samples and 3/48 (6.2%) were non-potable water samples. Sponge swab, stick swab and wastewater samples were compared for species and ARG recovery. There were no significant differences between the HH and VH sample types when number of species recovered was compared using the *t*-test (HH sponge mean = 71.8, standard deviation (SD) = 47.0, VH sponge mean = 52.1, SD = 94.9, HH wastewater mean = 232.6, SD = 140.6, VH wastewater mean = 93.8, SD = 98.6, HH stick mean = 88.2, SD = 32.8, VH stick mean = 35.7, SD = 48.8). Significantly more ARGs were recovered from the HH sponge swabs (mean = 37.3, SD = 36.1) compared with the VH sponge swabs (mean = 15.2, SD = 19.3) (*p* = 0.0080). There was no significant difference in ARG recovery between the HH and VH stick swabs (mean = 57.5, SD = 46.4 and mean = 17.3, SD = 12.5 respectively) or wastewater (mean = 55.9, SD = 36.6, mean = 19.2, SD = 24.3 respectively) (Figure 1).

### 2.2. Species

#### 2.2.1. HH Samples Show a Greater Species Variation than VH Samples

**Figure 2:**
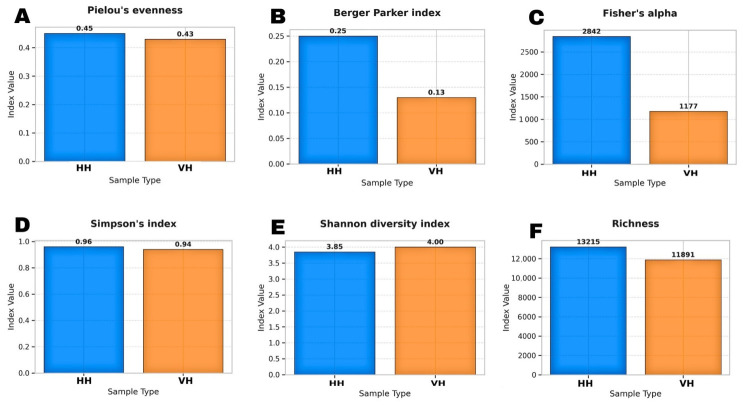
HH samples show a greater variation in species than VH samples. Box plots illustrating key alpha-diversity indices, including (**A**) Pielou’s evenness (community uniformity), (**B**) Berger–Parker index (dominance of the most abundant species), (**C**) Fisher’s alpha, (species richness), (**D**) Simpson’s index (probability of two randomly selected individuals belonging to the same species), (**E**) Shannon diversity index (species richness and evenness) and (**F**) Observed Richness (total number of distinct species). These tests demonstrate a distinct set of species, with the HH group showing greater species richness (**C**,**F**) and the VH group showing greater community complexity and balance.

Samples that contained at least one microbial genus (including bacteria, viruses, fungi, or protozoa) were included in the analysis. Overall microbial diversity was higher in the HH metagenomic samples when measured using the Shannon diversity index, which considers both the number of species and how evenly they are distributed. The results indicate that HH and VH samples have different microbial community structures. The HH sample had a much higher Fisher’s alpha and greater species richness, meaning they likely contained more distinct microbial species overall. By contrast, the VH samples had a slightly higher Shannon diversity index, suggesting their microbial communities may be more evenly balanced in terms of species abundance. The lower Berger–Parker index in the VH samples also indicates that no single species strongly dominated the communities compared with the HH group. Overall, HH samples showed greater dominance (Berger–Parker index) and higher estimated richness (Fisher’s alpha) (Figure 2).

#### 2.2.2. Distinct Microbial Communities Were Identified for HH and VH Samples

**Figure 3:**
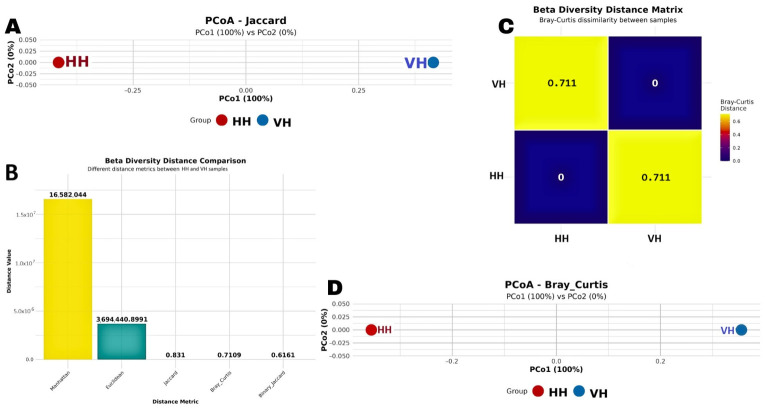
Beta-diversity analysis demonstrates significant differences in microbial community structure and composition between HH and VH groups. (**A**) PCoA based on Jaccard distances shows complete separation along PC1 (100% variation explained), indicating distinct communities. (**B**) Distance metrics (Manhattan, Euclidean, Jaccard, Bray–Curtis, Binary Jaccard) confirm large differences in community composition, both in the species present and their relative abundances. (**C**) Bray–Curtis dissimilarity matrix highlights pronounced differences between the samples within the HH and VH groups (value = 0.7). (**D**) PCoA using Bray–Curtis distances reaffirm clear separation between communities along PC1 (100% variation explained).

Principal Coordinates Analysis (Principal Coordinates Analysis, PCoA) using the Jaccard distance (Figure 3A) showed a complete separation between the HH and VH samples along the first principal coordinate (PCo1), which explained 100% of the variation. This indicates that the two samples contained very different sets of microbial taxa. A complementary assessment using Bray–Curtis dissimilarity, which also considers species abundance, produced a high dissimilarity value (0.7) (Figure 3C) between the HH and VH samples, confirming that differences extended beyond taxonomic presence to include major differences in their relative abundances.

Both samples exhibited zero within-group dissimilarity, as expected for single-sample comparisons, but the between-group difference indicates that the overall microbial compositions were strongly distinct. Comparative analysis of multiple distance metrics (Figure 3B) further supported this result. Manhattan and Euclidean distances showed the largest distances, reflecting substantial cumulative abundance differences between taxa; presence/absence metrics, including Jaccard and binary Jaccard, remained comparatively lower but still showed considerable separation. Bray–Curtis values were also high, confirming that differences in species abundance contributed strongly to the overall dissimilarity. Together, these results show that the HH and VH samples differed both in which taxa were present and in their abundance patterns. The Bray–Curtis PCoA plot (Figure 3D) mirrored the patterns observed with Jaccard distances. The HH and VH samples separated along PCo1, which explained 100% of the variation. The tight clustering and lack of overlap suggests very limited ecological similarity between the two environments.

Alpha-diversity analyses demonstrated that the HH samples had a higher richness (Fisher’s alpha, observed species), while the VH sample exhibited slightly higher Shannon diversity, indicative of a more even taxonomic distribution. However, these within-sample measures do not indicate similarity between communities. The pronounced beta-diversity separation indicated that the HH and VH samples contain different microbial communities overall, both in terms of taxa present and relative abundances. HH samples appear to contain more taxa but with greater dominance by one or a few species (reflected in the Berger–Parker index), whereas the VH samples are more evenly balanced without a strongly dominant taxon.

#### 2.2.3. There Was Multi-Kingdom Diversity in the VH Group Microbiome

**Figure 4:**
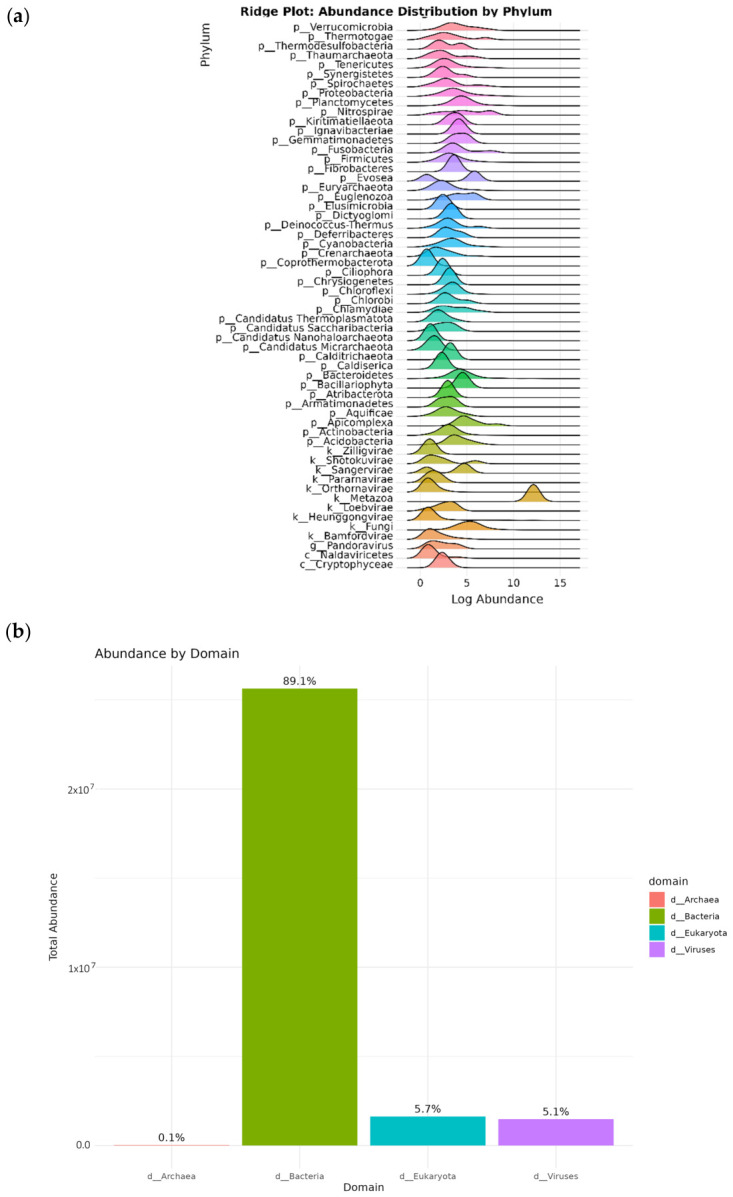
The VH group shows multi-kingdom diversity. (**a**) Ridge plot showing the distribution of log-transformed abundances across detected phyla, illustrating broad microbial diversity with dominant bacterial groups and lower contributions from archaeal, eukaryotic, and viral phyla. (**b**) Domain-level abundance summary, highlighting bacterial dominance (89.1%) alongside smaller proportions of eukaryota (5.7%), viruses (5.2%), and archaea (0.1%).

The taxonomic composition of the VH samples revealed a complex and highly diverse microbial community dominated by bacteria. The ridge plot (Figure 4a) displays the distribution of log-transformed abundances across all detected phyla, illustrating a wide range of evolutionary groups. Several bacterial phyla demonstrate clear abundance peaks. Bacteria accounted for 89.1% of total abundance (25,636,020 reads), reflecting their dominance across communities. Eukaryota (5.7%) and viruses (5.15%) form smaller but notable proportions, representing a mixture of fungal, protist, metazoan, and viral taxa, highlighting the multi-kingdom composition of the VH microbiome (Figure 4b). Archaea contributed 0.1% of total abundance (25,551 reads) (Figure 4a).

#### 2.2.4. HH Microbiomes Exhibited Less Diversity than VH Microbiomes

**Figure 5:**
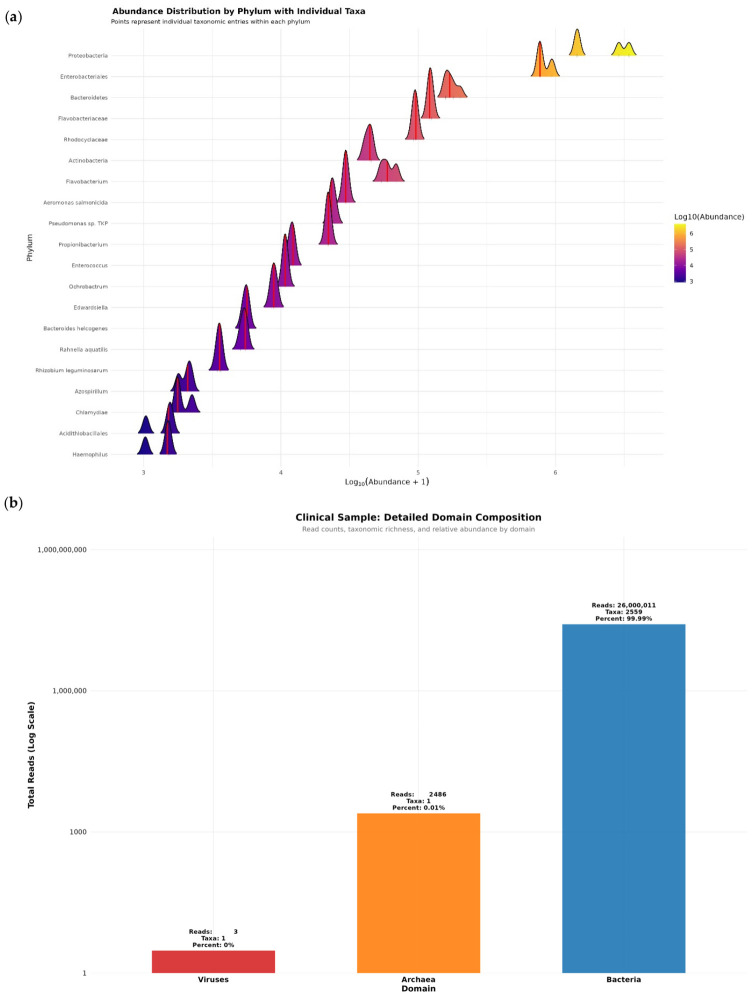
HH microbiomes exhibited less diversity than VH microbiomes. (**a**) The phylum-level distribution and relative abundances of dominant taxa in the HH microbiome, highlighting the strong presence of bacterial groups such as *Proteobacteria*, *Bacteroidetes*, and *Actinobacteria*. (**b**) The domain-level abundance profile, illustrating nearly total bacterial dominance with minimal archaeal and viral representation, reflecting the highly uneven community structure of the HH group.

The HH samples displayed strongly uneven microbial community structures dominated almost exclusively by bacteria. Figure 5a presents the abundance distribution of taxa within each phylum on a log scale, highlighting a small number of bacterial groups with very high abundance. Figure 5b summarizes the composition at the domain level. Bacteria constitute 26,000,011 reads, representing nearly 100% of the total classified sequences. Archaea were detected at very low levels (2486 reads; <0.01%), while viruses were nearly absent (3 reads; <0.01%). Beta diversity patterns showed clear separation of the HH samples, driven by the high abundance of clinically associated bacterial taxa and the minimal presence of archaeal or viral domains. Although the HH and VH samples shared some bacterial taxa, the HH community differed markedly in both abundance patterns and overall evenness.

#### 2.2.5. Notifiable, Reportable and Zoonotic Species Prevalence

The metagenomic samples were analyzed for notifiable human and veterinary pathogens, as well as the list of zoonotic diseases found in the UK [34,35,36]. Table 1 outlines the species found and the percentage of samples from the HH and VH samples they were found in. The VH samples had an average of 0.4 notifiable or zoonotic species (SD = 0.6), whilst the HH samples had an average of 0.9 notifiable/zoonotic species (SD = 1.2).

### 2.3. AMR

#### 2.3.1. Total Number of ARGs and Environmental Stressor Resistance Genes Are Higher in HH Samples

**Figure 6:**
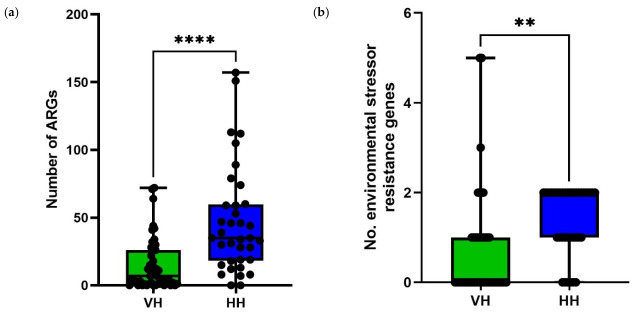
(**a**) Total number of ARGs (**** *p* < 0.0001) and (**b**) environmental stressor resistance genes (** *p* = 0.0010) identified per sample in HH and VH samples. There were more total ARGs and environmental stressor genes across HH samples compared with VH samples. Statistical analysis undertaken using Welch’s *t* test. Dots represent individual samples.

When the total number of ARGs was compared between the HH (mean = 47.4, SD = 39.4) and VH (mean = 15.0, SD = 18.9) samples, the HH samples showed significantly higher numbers (*p* < 0.0001), using Welch’s *t* test (Figure 6a). When the total number of environmental stressor resistance genes was compared between the HH (mean = 1.4, SD = 0.8) and VH (mean = 0.7, SD = 1.2) samples, the HH samples showed significantly higher numbers (*p* = 0.0010), using Welch’s *t* test (Figure 6b) [37,38].

ARGs and environmental stressor resistance genes were broken down by the class of antibiotic or stressor they caused resistance to. The highest number of individual/unique genes in class for VH were β-lactams (67 genes), aminoglycosides (44 genes) and tetracyclines (36 genes). For HH these were β-lactams (243 genes), aminoglycosides (91 genes) and sulfonamides (32 genes). The cumulative number of genes describes the number of individual genes multiplied by the number of times they were identified across all samples for VH and HH samples. For the VH samples, the highest cumulative number of genes in a class were the β-lactams (615 genes), aminoglycosides (479 genes) and sulfonamides (274 genes). For the HH samples, the highest cumulative number of genes in a class was the β-lactams (161 genes), aminoglycosides (130 genes) and sulfonamides (82 genes) (Table 2). Whilst HH samples often had the highest number of individual genes within a class, the VH samples had the highest number of cumulative genes within a class.

#### 2.3.2. Significantly Higher Numbers of High Risk ARGs Were Identified Across HH Samples

**Figure 7:**
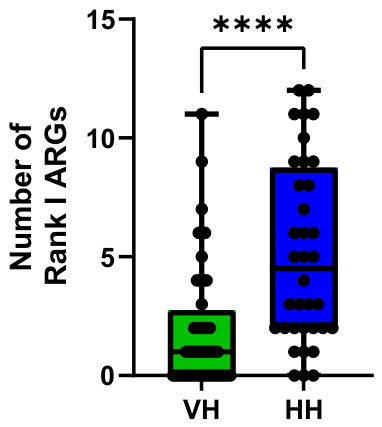
Comparison between the HH and environmental metagenomic samples for Rank I ARGs. The HH samples had significantly higher numbers of Rank I ARGs compared with the VH samples when an unpaired *t* test was used. **** = *p* < 0.0001. Dots represent individual samples.

High risk ARGs, as defined by Zhang et al. (2021), were compared using the *t*-test [39]. When total rank I ARGs were compared, there was statistical significance (*p* < 0.0001), with the HH samples having more total ARGs (mean = 5.1, SD = 3.8), than the VH samples (mean = 0.9, SD = 2.6). The HH samples also had significantly more Rank II ARGs (*p* = 0.0017), (mean = 0.8, SD = 0.8), compared with the VH samples (mean = 0.3, SD = 0.7) and significantly more Rank IV ARGs (*p* < 0.0001) (mean = 0.7, SD = 0.4). than the VH samples (mean = 0.2, SD = 0.4) (Figure 7).

For the VH samples, the mean number of ARGs was 1.9 (SD = 2.6). For the HH samples the mean number of ARGs was 5.1 (SD = 3.8). An overview of the number of samples each high risk ARG was identified in can be found in Table 3.

#### 2.3.3. More ARGs Were Unique to HH Samples

Of the total 592 ARGs present in the metagenomic analysis, 125 (21.1%) were unique to the VH environment and 377 (63.7%) were unique to the HH environment. A total of 90 (15.2%) were found in both sets of samples. Of the total 15 environmental stressor resistance genes identified, eight (53.3%) were unique to the VH samples and three (20.0%) were unique to the HH samples, with three (20.0%) being identified in both sets of samples. When this was broken down into classes, it was found that resistance genes for five antibiotic classes were uniquely found in the HH samples: colistin, fosfomycin, rifamycins, ‘multiple classes’ (antibiotics that had multiple functions) and efflux pumps. A total of 77.2% of the β-lactam genes identified were unique to the HH samples. Resistance genes for one environmental stressor gene class, for peroxide resistance, were also uniquely found in the HH samples. Resistance genes for one antibiotic class, the glycopeptides (bleomycin), were found to be unique to the VH samples (Table 4). Detailed resistance gene identifications for the HH and VH samples can be found in Elton et al. (2024) and Elton et al. (2025) [40,41].

### 2.4. Plasmid Composition

**Figure 8:**
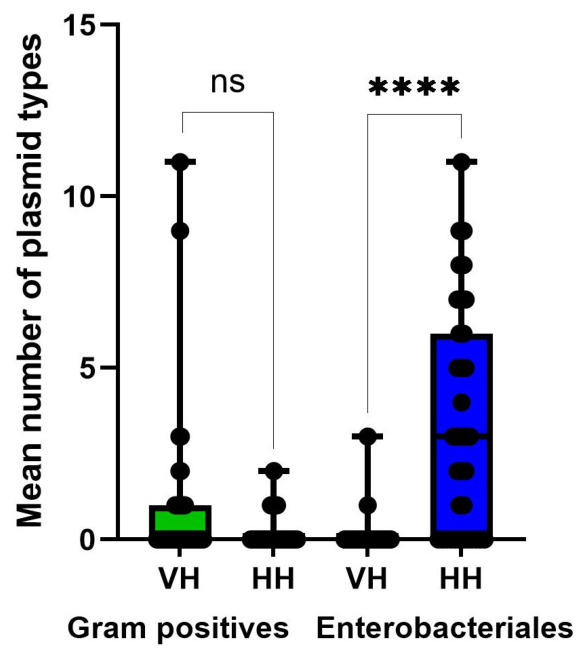
Mean number of Gram-Positive and Enterobacteriales plasmid types identified in the VH and HH samples. There was no significant difference between the HH and VH samples for Gram-Positive plasmid types, but the HH samples had significantly more Enterobacteriales plasmid types when compared with the VH samples (**** = *p* ≤ 0.0001) (Welch’s *t* test).

No significant difference was seen between the HH and VH samples for the Gram-Positive plasmid types. Significantly more Enterobacteriales plasmid types were found in the HH metagenomic samples (3.4 (SD = 3.3)) than the VH samples (0.1 (SD = 0.5)) (*p* < 0.0001). For an overview of plasmid type data, see Table 5. There was no significant difference in the number of Gram-Positive plasmid types found per sample when the HH samples were compared with the VH samples (Figure 8).

#### 2.4.1. Enterobacteriales Plasmid Types Were More Common in HH Samples

**Figure 9:**
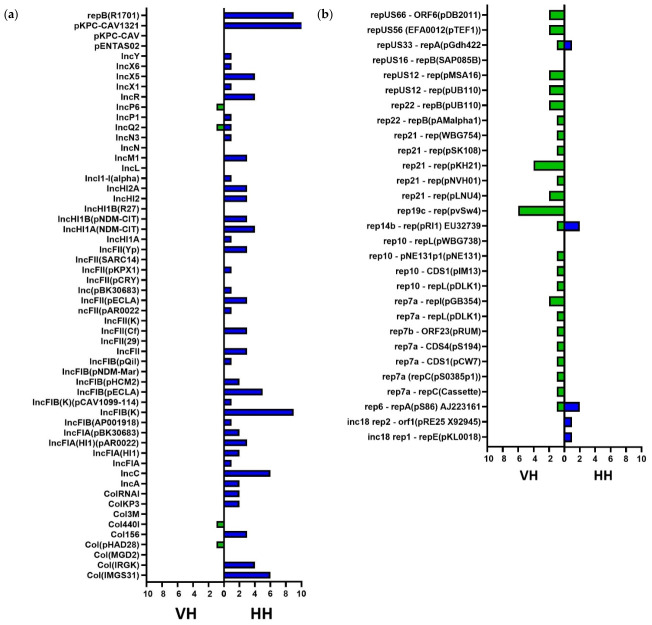
Comparison of (**a**) Enterobacteriales and (**b**) Gram-Positive plasmid types identified across the VH (green) and HH (blue) metagenomic samples.

There were significantly more total Enterobacteriales plasmids identified in the HH samples compared with the VH samples (*p* < 0.0001), and significantly more Enterobacteriales plasmid types were unique to the HH samples compared with the VH samples (*p* < 0.0001). A total of four Enterobacteriales plasmid types were identified from the VH samples, of which one (IncP6) was unique and not found in the HH samples. Enterobacteriales plasmid types were identified from 2/48 (4.2%) samples and the mean number of plasmid types per sample was 0.1 (SD = 0.5). The most common plasmid types were Col(pHAD28), Col440I, IncQ2 and IncP6 (one sample each) (Figure 9a). A total of 120 Enterobacteriales plasmid types were identified from the HH samples, of which 119 were unique. Enterobacteriales plasmid types were identified from 24/36 (66.7%) samples and the mean number of plasmid types per sample was 3.4 (SD = 3.3). The most common plasmid types were pKPC-CAV1321 (12 samples), IncFIB(K) and repB(R1701) (nine samples each). IncQ2 was the only plasmid type found in both the VH and HH samples.

#### 2.4.2. Gram-Positive Plasmid Types Were More Common in VH Samples

There were significantly more total Gram-Positive plasmids identified in VH samples compared with HH samples (*p* < 0.0001), and significantly more Gram-Positive plasmid types unique to the VH samples compared with the HH samples (*p* < 0.0001). A total of 40 Gram-Positive plasmid types were identified from the VH samples, of which 37 were unique and not found in the HH samples. Gram-Positive plasmid types were identified from 14/48 (29.2%) samples and the mean number of plasmid types per sample was 0.8 (SD = 2.1). The most common plasmid types were rep19c—rep(pvSw4) (six samples), rep21—rep(pKH21) (four samples), and rep7a—repI(pGB354), rep21—rep(pLNU4), rep22—repB(pUB110), repUS12—rep(pUB110), repUS12—rep(pMSA16), repUS56 (EFA0012(pTEF1) and repUS66—ORF6(pDB2011) (two samples each), all of which were unique to the VH samples (Figure 9b). For full plasmid details see [40,41].

A total of five Gram-Positive plasmid types were identified from the HH samples, of which two (inc18 rep1—repE(pKL0018) and inc18 rep2—orf1(pRE25 X92945)) were unique. Gram-Positive plasmid types were identified from 6/36 (16.7%) samples and the mean number of plasmid types per sample was 0.2 (SD = 0.5). The most common plasmid types were rep6—repA(pS86) AJ223161 and rep14b—rep(pRI1) EU32739 (two samples each). rep6—repA(pS86) AJ223161, rep14b—rep(pRI1) EU32739 and repUS33—repA(pGdh422) were all found in both the VH and HH samples.

## 3. Discussion

The HH and VH samples showed variation in the microbiomes, resistomes and plasmidomes identified, reflecting potential differences in HH and VH clinical practice and biology, but common themes were noted, highlighting IPC and surveillance implications across human and animal healthcare settings.

### 3.1. Microbiome

The diversity results indicate a greater number, and lower evenness, of species identified across the HH samples compared with the VH samples. This is perhaps unsurprising due to several factors, including selective pressures such as host immune responses and communal competition [42]. Human hospitals tend to have higher numbers of individuals entering the ward per day, including patients, healthcare workers and visitors. In the case of human hospitals, individuals within these sites are almost exclusively people (with the exception of, e.g., therapy animals); however, veterinary hospitals include several species, including humans, and therefore there is a greater chance of host species crossover [43]. Each individual brings their own microbiome, shedding bacteria and other organisms into the environment from their skin and hair, exhaled respiratory particles, clothing and footwear, and length of stay may affect the microbial load shed [44]. The greater the microbial introduction, the more species are likely to end up in the environment, and the routine use of disinfectants will affect the diversity of microbiomes in wastewater and on surfaces [45]. It is important to note that the sampled hospital ward was part of a tertiary hospital, so it was a larger institution than the companion animal veterinary hospital, which may account for some of the differences. It was also a single ward, rather than representative samples across the entire hospital, and intra-ward diversity is likely within both the HH and VH centres.

A greater variety of antimicrobial classes used in human hospitals, with more combination therapies and greater use of prophylactic antimicrobials [46]. This is likely to cause a greater disruption of patient microbiomes, the selection for resistant strains and a more rapid species turnover. This will result in a dynamic, constantly shifting microbial pool, increasing richness. The human tertiary hospital ward environment is also likely to be more complex than a companion animal veterinary hospital, with a greater number of medical devices and equipment, including more invasive items, each of which forms its own ecological niche to be filled by the microorganisms present [47].

As a result of this high richness but unevenness, fewer, more dominant pathogens could drive infections within the human hospital setting. Monitoring of the microbial community for shifts in composition could predict outbreak risks, and targeted IPC strategies should focus on these high-risk, hospital-adapted bacteria, which could include adaptive surface cleaning, hand hygiene and antibiotic stewardship. In veterinary settings a higher evenness and multi-kingdom diversity may suppress the dominance of specific pathogens via ecological competition. Contamination, including with MDROs, highlights that IPC is key, with the cornerstones of hand hygiene, cleaning and stewardship to minimize patient-to-patient transmission and zoonotic transmission in the context of the veterinary hospital.

The presence of sequencing reads for notifiable, reportable or zoonotic pathogens does not necessarily mean that live, culturable organisms are present, especially at a low percentage prevalence, as the majority of these were. DNA can persist in the environment and can be identified from lysed cells, extracellular DNA or environmental DNA fragments [48]. These samples were from a single time point at each site, so whilst detecting these organisms may appear concerning, the low copy number of these species may mean colonization may be transient, or there were no viable organisms in the samples. How these pathogens entered these environments would be a concern however, and if longitudinal sequencing identified an increase over time, it may suggest potential reservoirs. Of note was the fact that of the zoonotic species, only *Salmonella* and *Campylobacter* species were found across both the HH and VH samples, suggesting greater potential for OH transmission.

### 3.2. AMR

Compared with the VH samples, the HH samples had larger proportions of Rank I high risk ARGs, but as this ranking system is geared towards pathogens, and the ARGs found in them, in humans rather than animals, this may introduce bias [39]. This may also be partially explained by the difference in sizes between the tertiary HH and companion animal VH sampled. In both HH and VH settings in the UK, broad-spectrum β-lactams, including penicillins, β-lactam-inhibitor combinations, tetracyclines and macrolides are the most commonly prescribed antibiotics [49,50]. This study found that the resistomes across both the HH and VH samples commonly included β-lactam ARGs, with Rank I β-lactam ARGs especially prevalent in the HH samples. *mecA* was only found in the VH samples, which may be related to higher levels of staphylococci, in particular, *Staphylococcus pseudintermedius*, of which methicillin-resistant *S. pseudintermedius* is a known multi-drug-resistant (MDR) pathogen, particularly of dogs but occasionally of other species including humans [51]. That the majority of QAC resistance genes were identified in the VH samples may be concerning, as QAC products are often the main disinfectant used in companion animal practices in the UK.

Aminoglycoside ARGs were also commonly identified across both the HH and VH samples, although aminoglycoside use is usually restricted to resistant infections in both human and companion animal hospital settings [52,53]. Aminoglycoside resistance genes are however found across many Gram-Positive and Gram-Negative bacterial species, including non-pathogenic environmental ones [6,50]. The sulfonamides are not typically used as first-line antibiotics in human or companion animal practices, but *sul* genes, carried on plasmids and integrons, are also found in many bacterial species, including environmental ones, and species that can cause infection in humans and companion animals such as *Escherichia coli* and the streptococci [50,54,55].

The identification of high numbers of β-lactam, aminoglycoside and sulfonamide resistance genes, including high-risk ARGs, was notable from an IPC perspective. Enhanced environmental IPC procedures, especially the flushing out and disinfection of wastewater outlets may be necessary during outbreaks of high-risk AMR pathogens, such as the carbapenem-resistant Enterobacteriales (CRE). If an increase in multi-drug-resistant infections was seen within hospital settings, re-consideration of environmental cleaning, to reduce spread of these ARGs to potential nosocomial pathogens in patients, could be considered.

### 3.3. Plasmids

There was little overlap in plasmid types found across the HH and VH samples, which is perhaps not surprising given the relatively separate taxa found in each group. The VH samples had more Gram-Positive-associated plasmids, which may be due to the larger proportion of, and greater prevalence across samples, of *Staphylococci*, as described in greater detail in the previous literature [40,41]. Meanwhile, the HH samples had more plasmid types associated with Enterobacteriales, whose species are often associated with hospital-acquired species [56]. This is of concern for both human and veterinary settings, as whilst Gram-Positive plasmid types do carry ARGs, Enterobacteriales-associated ones are highly mobile, more easily transferred and more likely to carry high-risk ARGs such as extended spectrum β-lactamases (ESBLs), carbapenemases, and multidrug-resistance cassettes [57].

Any plasmids associated with AMR are a One Health issue and are capable of being spread between bacteria infecting both human and animal hosts and the environment [5]. The presence of both of these types of plasmids in both the HH and VH settings represents a transmission and infection risk to both humans and animals. A multidisciplinary approach would be needed to reduce these plasmids across patient and environmental settings, such as enhancing antimicrobial stewardship to reduce selection pressure, improving hygiene and sanitation to reduce the overall numbers of species and biomass present and reducing the opportunities for horizontal gene transfer by limiting ‘hotspots’ such as biofilm-heavy wastewater, shared equipment (and caging in the case of animals) [58]. The augmentation of hospital environments, such as ventilation and water safety measures, would also limit mixing of environmental species sources [59,60].

### 3.4. Importance of a Multisectoral Response

This study has highlighted both the similarities and differences between the microbiomes, resistomes and plasmidomes in a human and animal hospital setting and whilst these were individual sites, this adds to the voice from the previous literature, which highlights the need for a multisectoral approach towards the improved surveillance of AMR and pathogen transmission between patients and the environments that they are treated in [61]. Understudied but perhaps of particular importance in these potential transmission events are companion animals, and it is becoming increasingly clear that they may be significant reservoirs, especially when their human caregivers are immunocompromised or have had spells in hospital [62]. Equally, transmission goes both ways and reverse-zoonoses are increasingly being highlighted as a concern in the veterinary world [63].

### 3.5. Use of Metagenomics for Surveillance

Longitudinal environmental surveillance for ARGs and pathogens will be vital in high-risk settings such as human and animal hospitals to monitor changes in species prevalence and presence of ARGs. The One Health sharing of these data will help to elucidate intra- and inter-setting transmission risk. The use of metagenomics as a surveillance tool has advantages over other methods such as culture and molecular techniques such as PCR, as it can provide a complete, real-time and minimally biassed view of microbial communities. It provides greater resolution, identifies non-culturable organisms and provides genetic clues to uncover potential transmission risks. Whilst metagenomics may not be as sensitive for identifying specific factors, such as single base pair changes, as other methods, it can provide a holistic overview on microbial ecosystems without selection bias. The use of long read technologies such as Oxford Nanopore Technologies is advantageous, as reads may span many kilobases, helping to differentiate species, plasmids and ARGs. This is especially useful in environmental samples with multiple low biomass species.

Genomic surveillance is important for guiding a directed and truly One Health response, as it can pinpoint contamination hotspots (such as sinks, drains, equipment), identify high-risk wards or animal housing areas and environmental reservoirs and signal emerging high-risk clones. This can provide the evidence needed for IPC teams to undertake targeted action, such as enhanced cleaning of high-risk sites or the redesign of plumbing or water systems.

Whilst its strengths lay in community analysis and biome surveillance, metagenomics provides a lower sensitivity and resolution compared with whole genome sequencing. This may hinder the ability to uncover finer-scale relationships, such as determining which species harbour particular plasmids or extra-chromosomal AMR genes. The challenge is compounded by the rapid evolution of bioinformatics tools and databases used to detect these genetic features. As a result, distinguishing whether multidrug-resistant profiles originate from pathogenic organisms or from environmental or commensal species usually requires further ‘deep-dive’ analysis.

## 4. Methods

### 4.1. Sample Collection

All samples and data were obtained either from a large primary care companion animal veterinary hospital, or a tertiary referral and teaching hospital in London, UK.

### 4.2. HH Environmental Sampling

A site visit to the sampled ward was undertaken prior to collection, to evaluate layout and staff and patient routes of travel and sampling locations. Samples were taken from every multiple-occupancy-bed bay and single-bed room, covering areas where patients were known to have had a CRE, and non-affected areas. From each bay and room every sink and drain were sampled (in both bed and bathroom areas) and shower drains sampled. Non-clinical rooms were also covered, such as the shared-use pantry, staff toilets, nurses’ station, storage rooms, sluice, and workstations on wheels. Further details of sampling methodology, including types and locations, are described in Elton et al. (2024) [40].

### 4.3. VH Environmental Sampling

For environmental sampling, a site visit to the VH was undertaken prior to sample collection to evaluate room layout and staff and patient routes of travel and determine prioritized sampling locations including both patient and non-patient areas. Where there were multiple same-use rooms, e.g., consultation rooms, one representative location was chosen. Non-clinical rooms were also evaluated, including the visitor toilets. Samples were obtained on a normal working week afternoon shift in September 2022 from pre-designated locations. Sample types included wastewater samples (e.g., dog kennel waste drain), sponge swabs of surfaces (e.g., consulting tables), cotton stick swabs (e.g., sink drain holes) and liquid samples (e.g., ultrasound gel). Further details of sampling methodology, including types and locations, are described in Elton et al. (2025) [41].

### 4.4. DNA Extraction

DNA for metagenomic analysis was extracted directly from swab and water samples using the ZymoBIOMICS™ DNA Miniprep Kit (Zymo Research Corporation, Irvine, CA, USA), following the manufacturer’s instructions [64]. A sample of ZymoBIOMICS™ Microbial Community Standard (Zymo Research Corporation) was included, following the manufacturer’s instructions [65]. DNA quality was assessed for concentration using the Qubit™ dsDNA BR Assay Kit (Thermo Fisher, Waltham, MA, USA) and molecular weight and DNA integrity was confirmed using the Genomic DNA ScreenTape and reagents on the TapeStation 4150 (Agilent Technologies Inc., Santa Clara, CA, USA). Nuclease-free water (Sigma-Aldrich, St. Louis, MO, USA) was run as a negative control alongside samples from DNA extraction through to sequence analysis.

### 4.5. Library Preparation

Metagenomic DNA libraries were prepared using the ONT Rapid PCR Barcoding Kit (SQK-RPB004) with a DNA input of 1–5 ng and following the manufacturers’ instructions [66]. ZymoBIOMICS™ Microbial Community DNA Standards (Zymo Research Corporation) were included, following the manufacturer’s instructions [67].

### 4.6. Sequencing and Basecalling

Up to 24 barcoded metagenomic samples were run together on a flow cell version R9.4.1 (Oxford Nanopore Technologies, Oxford, UK) using a MinION device for 72 h, using the default parameters on the MinKNOW software (v23.04.6). Basecalling was performed either by the MinKNOW software alongside sequencing or using the Guppy basecalling software (v6.5.7) [68], using the flip-flop high accuracy algorithm, with a minimum Q score of 8.

### 4.7. Data Analysis

Of the 36 HH samples sequenced, all obtained reads. Of the 48 VH samples sequenced, nine did not yield any reads. Fastq files were quality checked using FastQC (v0.21.1) and MultiQC (v1.15) [69,70]. Sample data were analyzed for the presence of AMR genes using KmerResistance 2.2 (v02-2018), using the ResFinder AMR gene database (v4.2.5) and KmerFinder (v3.0.2), using the KmerFinder database (v2022-07-11), with 70% identity threshold, using the ONT pipeline [37,38]. Plasmids were identified using PlasmidFinder 2.1 (v2.0.1), using the PlasmidFinder database (v2023-01-18), with a minimum identity threshold of 95% and a minimum coverage threshold of 60% [38,71].

Species were assigned using Kraken (v2.1.3) and the NCBI Standard Refseq indexes PlusPF (v2022-06-07), which includes bacteria, viruses, fungi, archaea and the human genome [72,73]. Non-human mammalian genomes, including cat or dog genomes, were reported as unclassified. A confidence level of 0.1 was used when running Kraken2, as ONT data have lower quality scores than Illumina, and these were environmental samples.

### 4.8. Alpha and Beta Diversity

Taxonomic profiling was performed with Kraken2 (v2.1.3) using the PlusPFP-8 database spanning bacterial, archaeal, viral, fungal, protozoan, and plasmid genomes. Species-level read abundances were inferred with Bracken (v2.9). Bracken tables were imported into R (v4.3.2) via phyloseq (v1.44.0) and integrated with sample metadata. Alpha-diversity metrics (Richness, Shannon, Simpson, Pielou’s evenness, Berger–Parker, Fisher’s alpha) were computed using phyloseq and vegan (v2.6-4); group differences were evaluated with Wilcoxon rank-sum tests. Beta diversity was quantified using Bray–Curtis, Jaccard, Euclidean, and Manhattan distances (vegan::vegdist), followed by PCoA (vegan::capscale) and PERMANOVA (adonis2, 999 permutations). Visualizations, including boxplots and PCoA plots with 68% confidence ellipses, were produced with ggplot2 (v3.4.4). Statistical significance was set at *p* < 0.05.

### 4.9. Species Selection

The ‘reads (taxon)’ output from Kraken2 was used as the species read number, as the number of reads whose lowest common ancestor (LCA) is exactly that taxon. Taxa were discarded at <0.001–0.01% of total classified bases. Zoonotic species of interest were identified from the Public Health England (now UK Health Security Agency (UKHSA)) list of Zoonotic diseases of concern [36]. The UKHSA list of notifiable human pathogens and Department for Environment, Food and Rural Affairs (DEFRA) and the Animal and Plant Health Agency (APHA) list of notifiable veterinary pathogens were used to select species of interest from the metagenomic samples [34,35].

### 4.10. Selection of High-Risk ARGs

Based on the health-risk ARGs framework, 122 high-risk ARGs were screened, of which 72 were unique and Rank I, based on the ranking system of Zhang et al. (2021) [31,39]. Additional environmental stressor genes: *sit* (peroxide resistance), *qac* (increased tolerance to quaternary ammonium compounds decreased and other cationic biocides such as chlorhexidine) and *cpl* (heat resistance) were also identified to assess environmental persistence, as part of the KmerResistance 2.2 database.

### 4.11. Statistical Analysis

Statistical analysis was conducted using Prism v10.3.0 (GraphPad). The test used for each analysis is described within the Results Section. Sequence data were deposited in European Nucleotide Archive (ENA) under BioProject PRJEB84924 (VH samples) and PRJEB76684 (HH samples) and are outlined in the Supplementary Materials sections of references [40,41]. For analysis of HH isolate AMR status, an isolate was deemed MDR if it showed resistance to antibiotics in three or more antibiotic classes, to which the bacterial species does not show intrinsic resistance [74,75].

## Figures and Tables

**Table 1:** List of pathogenic species identified from HH and VH metagenomic samples that were either notifiable human or veterinary pathogens, and/or on the UK zoonotic diseases list (denoted by ✓). * *Brucella* is ‘reportable’ in dogs and cats but ‘notifiable’ in other species. ** Notifiable, if associated with food poisoning, but required to report in veterinary medicine.

Disease	Organism	Zoonotic List	Notifiable Human	Notifiable Veterinary	VH		HH	
					Number of Samples Present	Percentage Prevalence	Number of Samples Present	Percentage Prevalence
Kennel cough	*Bordetella bronchiseptica*	✓			0/48 (0.0%)	n/a	1/36 (2.8%)	0.04
Brucellosis	*Brucella* spp. *	✓	✓	✓	0/48 (0.0%)	n/a	4/36 (11.1%)	0.01–0.10
Glanders	*Burkholderia mallei*	✓	✓	✓	0/48 (0.0%)	n/a	1/36 (2.8%)	0.02
Campylobacteriosis	*Campylobacter* spp.	✓	✓		3/48 (6.3%)	0.01–0.10	7/36 (19.4%)	0.02–0.05
Salmonellosis **	*Salmonella* spp.	✓	✓	✓	1/48 (2.1%)	0.04	4/36 (11.1%)	0.01–0.02

**Table 2:** Breakdown of ARGs in relation to the antibiotic and environmental stressor class they cause resistance to. Total individual genes in class describes the number of unique genes causing resistance to that class found across all samples for VH and HH samples. The cumulative number of genes describes the number of individual genes, multiplied by the number of times they were identified across all samples for VH and HH samples. ESR = environmental stressor resistance. Total individual genes in class describes the number of unique genes causing resistance to that class found across all samples for VH and HH samples. The colour scale represents increasing numbers (green = low, to red = high).

		Total Individual Genes in Class		Cumulative Number of Genes	
	Resistance Against Antibiotic Class	VH	HH	VH	HH
Antibiotic resistance class	Aminoglycosides	44	91	479	130
	β-lactams	67	243	615	161
	Amphenicols	7	8	14	7
	Polymyxin (colistin)	0	3	8	0
	Phosphonic acid antibiotics (fosfomycin)	0	2	7	0
	Glycopeptide antibiotics (bleomycin)	1	0	0	3
	Lincosamides	4	7	8	18
	Macrolides	4	7	17	12
	Erythromycins	16	8	47	32
	Fluoroquinolones	3	10	25	4
	Rifampicin	0	1	2	0
	Streptogramin A and related antibiotics	1	1	1	1
	Sulfonomides	28	32	274	84
	Tetracyclines	32	20	68	57
	Trimethoprims	6	25	69	12
	Efflux pump	0	2	11	0
ESR	Peroxide resistance	0	1	1	0
	Decreased susceptibility to chlorhexidine	10	2	48	31
	Heat and stress resistance	1	3	3	1

**Table 3:** High risk ARGs and the number of samples that resistance genes were found in for the VH and HH samples.

Gene	VH	HH	Gene	VH	HH
Aminoglycosides			Macrolide–Lincosamide–Streptogramin Group		
*aac(3)-II*	2/48 (4.2%)	14/36 (38.9%)	*ermB*	5/48 (10.4%)	0/36 (0.0%)
*aac(3)-VI*	2/48 (4.2%)	0/36 (0.0%)	*ermC*	7/48 (14.6%)	0/36 (0.0%)
*aac(6′)-I*	0/48 (0.0%)	1/36 (2.8%)	*ermT*	0/48 (0.0%)	1/36 (2.8%)
*ant(2″)-I*	1/48 (2.1%)	16/36 (44.4%)	*lnuA*	11/48 (22.9%)	1/36 (2.8%)
*aph(3′)-I*	9/48 (18.8%)	23/36 (63.9%)	*mphA*	0/48 (0.0%)	3/36 (8.3%)
*aph(6)-I*	16/48 (33.3%)	23/36 (63.9%)	Multidrug		
β-lactam			*emrB-qacA*	3/48 (6.3%)	0/36 (0.0%)
*blaZ*	5/48 (10.4%)	0/36 (0.0%)	Quinolones		
*bla*_CMY-6_	0/48 (0.0%)	1/36 (2.8%)	*qnrA*	0/48 (0.0%)	2/36 (5.6%)
*bla*_CTX-M-15_	0/48 (0.0%)	6/36 (16.7%)	*qnrB*	0/48 (0.0%)	13/36 (36.1%)
*bla*_CTX-M-55_	0/48 (0.0%)	2/36 (5.6%)	*qnrS*	2/48 (4.2%)	4/36 (11.1%)
*mecA*	5/48 (10.4%)	0/36 (0.0%)	Tetracyclines		
*bla*_OXA-1_	0/48 (0.0%)	12/36 (33.3%)	*tetL*	1/48 (2.1%)	0/36 (0.0%)
*bla*_OXA-10_	0/48 (0.0%)	7/36 (19.4%)	*tetM*	1/48 (2.1%)	0/36 (0.0%)
*bla*_SHV-5_	0/48 (0.0%)	1/36 (2.8%)	Trimethoprim		
*bla*_TEM-1_	11/48 (22.9%)	11/36 (30.6%)	*dfrA1*	5/48 (10.4%)	9/36 (25.0%)
*bla*_TEM-156_	3/48 (6.3%)	0/36 (0.0%)	*fdfrA5*	0/48 (0.0%)	17/36 (47.2%)
*bla*_VIM-1_	0/48 (0.0%)	2/36 (5.6%)	*dfrA12*	0/48 (0.0%)	3/36 (8.3%)
*bla*_VIM-2_	0/48 (0.0%)	2/36 (5.6%)	*dfrA14*	0/48 (0.0%)	3/36 (8.3%)
Chlorampenicol			*dfrA17*	0/48 (0.0%)	1/36 (2.8%)
*catA*	1/48 (2.1%)	4/36 (11.1%)			
*catB*	2/48 (4.2%)	0/36 (0.0%)			
*cmlA*	0/48 (0.0%)	1/36 (2.8%)			

**Table 4:** Number of ARGs conferring resistance to each antibiotic class and against environmental stressors that were unique to the VH metagenomic samples and HH metagenomic samples and that were present in both. * Streptogramin A and related antibiotics. The percentage is of the total genes identified for each class. QAC = quaternary ammonium compound.

	Genes Unique to VH Samples	Genes Unique to HH Samples	Genes Present in Both
Antibiotic Class			
Aminoglycosides	17/103 (16.5%)	57/103 (55.3%)	29/103 (28.2%)
β-lactams	52/295 (17.6%)	228/295 (77.3%)	15/295 (5.1%)
Amphenicols	4/12 (33.3%)	5/12 (41.7%)	3/12 (25.0%)
Polymyxin (colistin)	0/3 (0.0%)	3/3 (100%)	0/3 (0.0%)
Phosphonic acid antibiotics (fosfomycin)	0/2 (0.0%)	2/2 (100%)	0/2 (0.0%)
Glycopeptide antibiotics (bleomycin)	1/1 (100%)	0/1 (0.0%)	0/1 (0.0%)
Lincosamides	3/10 (30.0%)	6/10 (60.0%)	1/10 (10.0%)
Macrolides	2/9 (22.2%)	5/9 (55.6%)	2/0 (22.2%)
Erythromycins	14/22 (63.6%)	6/22 (27.3%)	2/22 (9.1%)
Fluoroquinolones	1/11 (9.1%)	8/11 (72.7%)	2/11 (18.2%)
Rifamycins	0/1 (0.0%)	1/1 (100%)	0/1 (0.0%)
Streptogramin A *	1/2 (50%)	1/2 (50%)	0/2 (0.0%)
Sulfonomides	5/47 (10.6%)	19/47 (40.4%)	23/47 (48.9%)
Tetracyclines	24/44 (54.5%)	12/44 (27.3%)	8/44 (18.2%)
Trimethoprim	1/26 (3.8%)	20/26 (76.9%)	5/26 (19.2%)
Multiple classes	0/2 (0.0%)	2/2 (100%)	0/2 (0.0%)
Efflux pumps	0/2 (0.0%)	2/2 (100%)	0/2 (0.0%)
Environmental stressors			
Peroxide resistance	0/1 (0.0%)	1/1 (100%)	0/1 (0.0%)
QAC disinfectants	8/10 (80.0%)	0/10 (0.0%)	2/10 (20.0%)
Heat and stress resistance	0/3 (0.0%)	2/3 (66.7%)	1/3 (33.3.%)
Total (antibiotic classes)	125/592 (21.1%)	377/592 (63.7%)	90/592 (15.2%)
Total (environmental stressors)	8/14 (57.1%)	3/14 (21.4%)	3/14 (21.4%)
Total (both)	133/606 (21.9%)	380/606 (62.7%)	93/606 (15.3%)

**Table 5:** Overview of plasmids found. Statistical analysis undertaken using Welch’s *t* test.

	Enterobacteriales			Gram Positives		
	VH	HH	Significance	VH	HH	Significance
Mean number of plasmid types per samples (SD)	0.1 (SD = 0.5)	3.4 (SD = 3.3)	*p* < 0.0001	0.8 (SD = 2.1)	0.2 (SD = 0.5)	No significance
No. samples that plasmid types identified from (%)	2/48 (4.2%)	24/36 (66.7%)	n/a	14/48 (29.2%)	6/36 (16.7%)	n/a
Total plasmid types identified	4	120	*p* < 0.0001	40	5	*p* < 0.0001
Number of unique plasmid types	3	119	*p* < 0.0001	35	2	*p* < 0.0001
Number of plasmid types in both	1		n/a	3		n/a

## Data Availability

All data is Open Access. All data is Open Access and is available on the European Nucleotide Archive, under the project accession numbers PRJEB84924 (VH data) and PRJEB76684 (HH data).

## Abbreviations

AMR	Antimicrobial resistance
ARGs	Antimicrobial resistance genes
CRE	Carbapenem-resistant Enterobacteriales
HH	Human hospital
IPC	Infection prevention and control
MDR	Multi-drug resistance
MDRO	Multi-drug-resistant organism
OH	One Health
ONT	Oxford Nanopore Technologies
PBS	Phosphate-buffered saline
PCR	Polymerase chain reaction
SD	Standard deviation
VH	Veterinary hospital
